# Animals’ Diseases and Man’s

**Published:** 1898-09

**Authors:** 


### ANIMALS' DISEASES AND MAN'S.

The fact that so many of the diseases affecting animals are communicated to man has largely been the means of bringing animal diseases within range of scientific attention. That so much is suffered in common makes animals the objects of scientific symathy. If the measures of inspection recommended by the United States Department of Agriculture in their recent bulletin be generally adopted, many of the diseases derived solely from the lower animals, such as glanders, hydatid disease, trichinosis, and similar affections, are likely to be stamped out.

By consideration of the troubles that come to us from the animal world a proper respect is engendered for the general healthiness which happily prevails in the animal kingdom. Animals are, without doubt, frequently sick, oftener so than is commonly believed. Tuberculosis, it is known, is widely prevalent among domestic species, especially those used for flesh-food purposes. Koch's claim, however, of the unity of the tubercle bacillus in all animals has not been safely established. The disease is probably most common with animals which have come in touch with civilization, though it is not restricted to them. The domestic animals suffer many complaints, yet gratitude is due them that in their bills of mortality the principal cause of death must figure as violence due to the predatory instinct of man.

Among wild animals disease is probably rarer. Naturalists have never yet fairly answered the question: Of what do animals die? It has been conjectured that starvation is the principal cause, but it is doubtful if this cause is often epidemic. In the arctic regions, where life during the short summer is numbered by millions, and where there is a larger number of birds and large and small animals gathered together than in any other part of the globe, the remains of dead animals are said never to be found, notwithstanding the severity of the winter which is always at their heels. How these animals die is a mystery. In Spitzbergen it is said to be easier to find the vertebræ of a gigantic lizard of the Trias than the remains of a seal, walrus or bird which has met a natural death. Wild animals, however, do die. Tuberculosis, as a cause of death, is widely prevalent. Anthrax is a deadly and rapid cause of death. Influenza is nearly always present and often epidemic. Animals may die of anything from malignant tumor to heart disease. Mr. George Fleming, in his interesting book on "Animal Plagues," enumerates eighty-six epidemics affecting wild quadrupeds and birds and twenty-seven affecting fish. There is, indeed, good reason to believe that epidemic disease is very prevalent among wild species, and that it is the usual cause of death among those who escape starvation.—*Medical Age.*

